# Preoperative predictive model for the probability of lymph node metastasis in gastric cancer: a retrospective study

**DOI:** 10.3389/fonc.2024.1473423

**Published:** 2024-09-27

**Authors:** Fei Teng, Qian Zhu, Xi-Lang Zhou, Yi-Bing Shi, Han Sun

**Affiliations:** ^1^ Department of Interventional Radiology, The First Affiliated Hospital of Ningbo University, Ningbo, China; ^2^ Department of Radiology, Xuzhou Central Hospital, Xuzhou, China; ^3^ Department of Gastroenterology, Xuzhou Central Hospital, Xuzhou, China

**Keywords:** computed tomography, lymph node, metastasis, gastric cancer, prediction

## Abstract

**Background:**

Effectively diagnosing lymph node (LN) metastasis (LNM) is crucial in determining the condition of patients with gastric cancer (GC). The present study was devised to develop and validate a preoperative predictive model (PPM) capable of assessing the LNM status of individuals with GC.

**Methods:**

A retrospective analysis of consecutive GC patients from two centers was conducted over the period from January 2021 to December 2023. These patients were utilized to construct a 289-patient training cohort for identifying LNM-related risk factors and developing a PPM, as well as a 90-patient testing cohort used for PPM validation.

**Results:**

Of the GC patients included in the training cohort, 67 (23.2%) and 222 (76.8%) were respectively LNM negative and positive. Risk factors independently related to LNM status included cT3 invasion (P = 0.001), CT-reported LN (+) (P = 0.044), and CA199 value (P = 0.030). LNM risk scores were established with the following formula: score = -2.382 + 0.694×CT-reported LN status (+: 1; -: 0)+2.497×invasion depth (cT1: 0; cT2: 1; cT3: 2)+0.032×CA199 value. The area under the curve (AUC) values for PPM and CT-reported LN status were 0.753 and 0.609, respectively, with a significant difference between them (P < 0.001). When clinical data from the testing cohort was included in the PPM, the AUC values for the PPM and CT-reported LN status were 0.756 and 0.568 (P < 0.001).

**Conclusions:**

The established PPM may be an effective technique for predicting the LNM status of patients preoperatively. This model can better diagnose LNM than CT-reported LN status alone, this model is better able to diagnose LNM.

## Introduction

Lymph node (LN) metastasis (LNM) is a crucial clinical finding in gastric cancer (GC) patients that is related to TNM staging, therapeutic planning, and prognostic outcomes (1–4). The choice of suitable surgical techniques can be influenced by a patient’s LNM status (5–8). Patients with early T-stage GC who are LNM negative have a more limited range of surgical resection options than those who are LNM positive. Neoadjuvant chemotherapy is also commonly administered to LNM-positive GC patients in an attempt to decrease preoperative staging and increase the likelihood of successful radical resection (8, 9). Therefore, it is advantageous to precisely determine the LNM status of a GC patient before their surgical procedure.

Currently, the evaluation of LNM status in GC predominantly decided by the size of the LN using computed tomography (CT) (4). Although the size of a target node can provide good specificity (86%) when assessing LNM status in this patient population, its sensitivity levels are substantially lower (67%) (4). To improve the accuracy of predicting LNM status, multiple research has investigated shifting focus from conventional imaging features in favor of radiomics techniques (10–15). As radiomics strategies tend to lack reproducibility and are difficult to standardize across software tools, however, they have not been used widely in clinical settings to date (1).

Unlike radiomics techniques, traditional PPMs are usually developed based on the clinical and normal image data. The traditional clinical PPM has some advantages: (a) all clinical and normal image data can be directly obtained from the Hospital Information System (HIS) and Picture Archiving and Communication Systems (PACS); (b) all clinical and normal image data can be directly used without needing data transform; (c) the clinical and normal image data have the unified criterion and these data can be easily understood. Individual clinical or image variable may difficult to exhibit a high diagnostic accuracy. However, combining many variables together as a PPM can provide a well diagnostic performance.

This study was designed to develop and validate a preoperative predictive model (PPM) for determining the LNM status of patients diagnosed with GC.

## Materials and methods

### Study design

This retrospective analysis was approved by the Ethics Committee of The First Affiliated Hospital of Ningbo University and Xuzhou Central Hospital and the requirement for informed consent was waived. A training cohort consisting of 289 consecutive patients with GC from Xuzhou Central Hospital between January 2021 and December 2023 was established. Furthermore, a testing cohort comprising 90 consecutive GC patients from The First Affiliated Hospital of Ningbo University during the same period was established. Data from the training cohort were utilized to establish the PPM, and the same data from the training cohort were used to validate the PPM.

Identical inclusion and exclusion criteria were applied to the training and validation cohorts. To be considered for inclusion, patients had to meet the following criteria: (1) they must have received a diagnosis of GC based on a preoperative gastroscopy, (2) their GC must be of the adenocarcinoma GC pathological type, (3) they must have undergone serum testing, gastroscopy, and CT scanning within 7 days before surgical resection, (4) they must have undergone gastrectomy with D2 lymphadenectomy, and (5) their LNM status must have been confirmed through pathological examination. Patients were excluded if they (1) lacked complete clinical data needed for this study, (2) exhibited any other comorbid tumors, or (3) had undergone preoperative antitumor treatment.

The data utilized for these analyses encompassed age, gender, tumor size, tumor location, depth of invasion, CT-based LN status, pathological findings, and the results of tumor marker tests.

### CT scanning

A 64 Dual-Source CT instrument was used for all CT scanning. Patients were directed to fast for >8 hours before scanning, and received an intravenous injection of anisodamine (20 mg) to prevent gastric motility. Patients also orally consumed 1 L of warm water before scanning for stomach dilation, and were directed to hold their breath during scanning. Both non-enhanced and enhanced CT scanning were performed as detailed in the **Supplementary Materials**. Two radiologists with 8 and 15 years of expertise interpreting abdominal CT pictures independently assessed the resulting images, keeping the patient’s pathological diagnosis confidential. A third radiologist with 20 years of experience interpreting abdominal diagnosis resolved any disagreements. CT-reported LN positivity was determined based on a short axial diameter for the target LN exceeding 5 mm.

### Gastroscopy

During the gastroscopy, information was obtained on the tumor’s location, tumor size, and presence or absence of ulcers. The depth of tumor invasion [clinical T stage (cT stage)] was determined through endoscopic ultrasonography. Every patient in the research population had GC that was verified by biopsy pathology. Moreover assessed were the tumor’s pathological type and differentiation.

### PPM development and validation

Through univariate and multivariate logistic regression analyses that evaluated clinical features, CT results, gastroscopy findings, and the amounts of investigated tumor markers. LNM-related risk variables were identified in the training cohort and then the PPM was established based on the risk variables. Data from the testing cohort was then used for PPM validation.

### Statistical analyses

SPSS 27.0 and R 4.1.2 were used for all analyses. Categorical data were compared using χ^2^ tests or Fisher’s exact test. Continuous data that were normally (non-normally) distributed were compared using Student’s t-tests (Mann-Whitney U tests). LNM-related risk factors were selected through logistic regression analyses. The PPM was established based on the LNM-related risk factors and a risk scoring formula was listed. The nomogram was constructed based on the risk scoring formula by using the “rms” R package based on the training cohort. Comparisons of area under the ROC curve (AUC) values were made with the DeLong test. The inter-observer agreement analyzes were conducted with Intraclass Correlation Coefficient test for continuous data or Kappa analysis for categorical data. The Intraclass Correlation Coefficient or Kappa values were used to assess the degree of agreement (< 0.21, poor; 0.21–0.4, fair; 0.41–0.6, moderate; 0.61–0.8, good; and 0.81–1, very good).

## Results

### Patients characteristics

Of the GC patients in the training cohort for this study, 67 (23.2%) and 222 (76.8%) were respectively negative and positive for LNM (**Table 1**). In LNM positive group, patients exhibited significantly higher rates of cT3 invasion, the presence of ulcers, moderate/poor differentiation, and CT-reported LN-positive status than those in the LNM negative group. Furthermore, LNM positive group also exhibited significantly larger tumor diameter and higher CEA value than LNM negative group. No other analyzed parameters differed between patients who were and were not LNM positive in this training cohort. The inter-observer agreements of maximum tumor diameter and CT-reported LN status were very good in both training and test cohorts (**Supplementary Table 1**).

**Table 1:** Patients baseline data.

	Training cohort			Test cohort			P inter-groups
	LNM (-)	LNM (+)	P	LNM (-)	LNM (+)	P	
Patients number	67	222		29	61		
Age (year)	66.7 ± 10.0	65.4 ± 10.0	0.357	65.6 ± 9.0	67.9 ± 8.6	0.260	0.230
Sex			0.288			0.963	0.859
Male	48	173		22	46		
Female	19	49		7	15		
Tumor location			0.361			0.984	0.157
Preventriculus	10	46		8	19		
Fundus	9	17		3	6		
Body	27	80		10	21		
Pylorus	21	77		8	15		
Maximum tumor diameter (mm)	38.6 ± 19.9	50.0 ± 22.8	< 0.001	34.2 ± 17.9	56.7 ± 25.1	< 0.001	0.461
Tumor invasion depth			0.001			0.001	0.517
cT1	15	4		7	0		
cT2	11	11		6	4		
cT3	41	207		16	57		
CT-reported LN (+)			0.002			0.202	0.014
Yes	21	118		7	23		
No	46	104		22	38		
Ulcer			0.001			0.004	0.341
Yes	42	182		19	55		
No	25	40		10	6		
Combined ring cell carcinoma			0.100			0.783	0.193
Yes	12	62		5	12		
No	55	160		24	49		
Differentiation			0.008			0.002	0.255
Well	4	9		1	0		
Moderate	29	55		13	9		
Poor	34	158		15	52		
CEA value (ug/L, normal range: 0-5 ug/L)	1.6 (0.9; 2.5)	1.9 (1.1; 5.0)	0.032	1.9 (0.9; 2.3)	1.8 (1.2; 6.0)	0.110	0.999
CA199 value (kU/L, normal range: 0-37 kU/L)	8.5 (4.6; 11.9)	9.6 (4.6; 20.8)	0.125	7.5 (4.7; 12.2)	9.3 (3.8; 21.6)	0.322	0.737
CA125 value (U/ml, normal range: 0-35 U/ml)	6.9 (4.4; 14.4)	7.5 (4.3; 12.8)	0.791	6.9 (5.7; 13.2)	7.8 (4.6; 10.7)	0.779	0.914

CA125, carbohydrate antigen-125; CA199, carbohydrate antigen-199; CEA, carcinoembryonic antigen; CT, computed tomography; cT, clinical T stage; LN, lymph node; LNM, LN metastasis.

### LNM-related risk factors

In univariate analyses, tumor location at the fundus (P = 0.084), maximum tumor diameter (P = 0.001), cT2 invasion (P = 0.061), cT3 invasion (P = 0.001), CT-reported LN (+) (P = 0.026), the presence of ulcers (P = 0.001), CEA value (P = 0.077), and CA199 value (P = 0.021) were significantly related to LNM. The tumor diameter, CEA value, and CA199 value were positively correlated with LNM. In multivariate analyses, cT3 invasion (P = 0.001), CT-reported LN positivity (P = 0.044), and CA199 value (P = 0.030) were found to be independently associated with LNM risk. The CA199 value was positively correlated with LNM (**Table 2**).

**Table 2:** Risk factors of LNM.

	Univariate analysis			Multivariate analysis		
	Hazard ratio	95% CI	P value	Hazard ratio	95% CI	P value
Age (year)	0.987	0.959-1.015	0.356			
Sex						
Male	1					
Female	0.716	0.385-1.328	0.716			
Tumor location						
Preventriculus	1			1		
Fundus	0.349	0.137-1.132	0.084	0.462	0.144-1.480	0.193
Body	0.617	0.275-1.386	0.242	0.722	0.284-1.835	0.494
Pylorus	0.764	0.331-1.760	0.527	1.008	0.394-2.581	0.986
Maximum tumor diameter (mm)	1.028	1.013-1.045	0.001	1.009	0.993-1.024	0.277
Tumor invasion depth						
cT1	1			1		
cT2	3.750	0.940-14.963	0.061	4.275	0.905-20.205	0.067
cT3	18.933	5.979-59.955	0.001	12.143	2.958-49.848	0.001
CT-reported LN (+)						
No	1			1		
Yes	1.931	1.081-3.447	0.026	2.001	1.020-3.925	0.044
Ulcer						
No	1			1		
Yes	2.708	1.484-4.944	0.001	1.725	0.788-3.777	0.172
Combined with ring cell carcinoma						
No	1					
Yes	1.776	0.891-3.540	0.103			
Differentiation						
Well	1					
Moderate	0.843	0.239-2.974	0.790			
Poor	2.065	0.601-7.099	0.250			
CEA value (ug/L)	1.033	0.997-1.070	0.077	1.011	0.984-1.039	0.420
CA199 value (kU/L)	1.031	1.005-1.058	0.021	1.033	1.003-1.063	0.030
CA125 value (U/ml)	1.003	0.992-1.014	0.602			

CA125, carbohydrate antigen-125; CA199, carbohydrate antigen-199; CEA, carcinoembryonic antigen; CT, computed tomography; cT, clinical T stage; LN, lymph node; LNM, LN metastasis.

### PPM development

**Figure 1:**
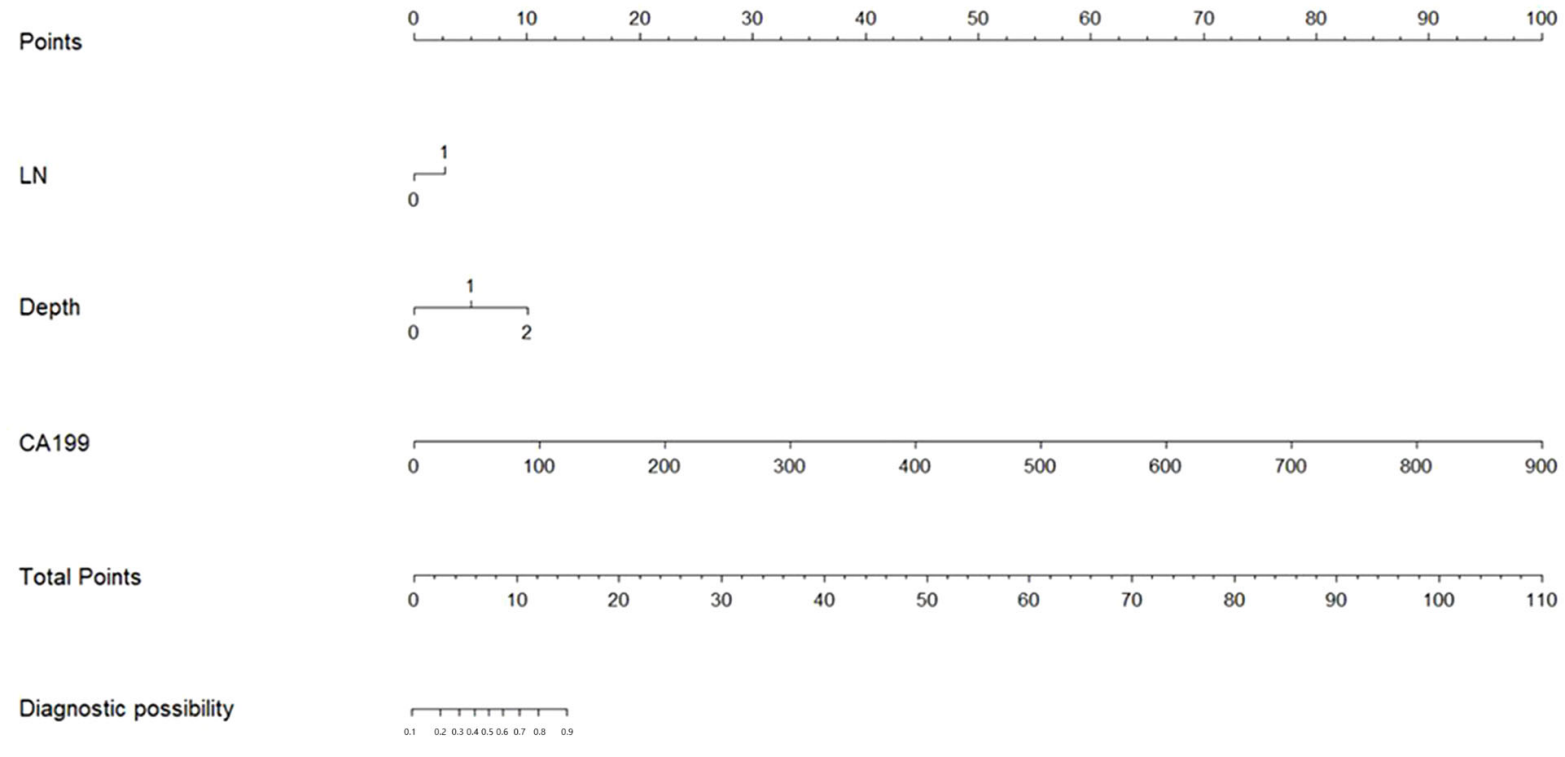
The nomogram of this predictive model.

The risk factors identified above were used to establish a PPM and nomogram (**Figure 1**). The risk scoring formula for the nomogram was the following: score = -2.382 + 0.694×CT-reported LN status (+: 1; -: 0)+2.497×invasion depth (cT1: 0; cT2: 1; cT3: 2)+0.032×CA199 value. The cut-off score of 3.4217 demonstrated the highest sensitivity (52.3%) and specificity (86.6%), Patients were classified as LNM positive if their score at or above this threshold value, while they were otherwise classified as LNM negative.

**Figure 2:**
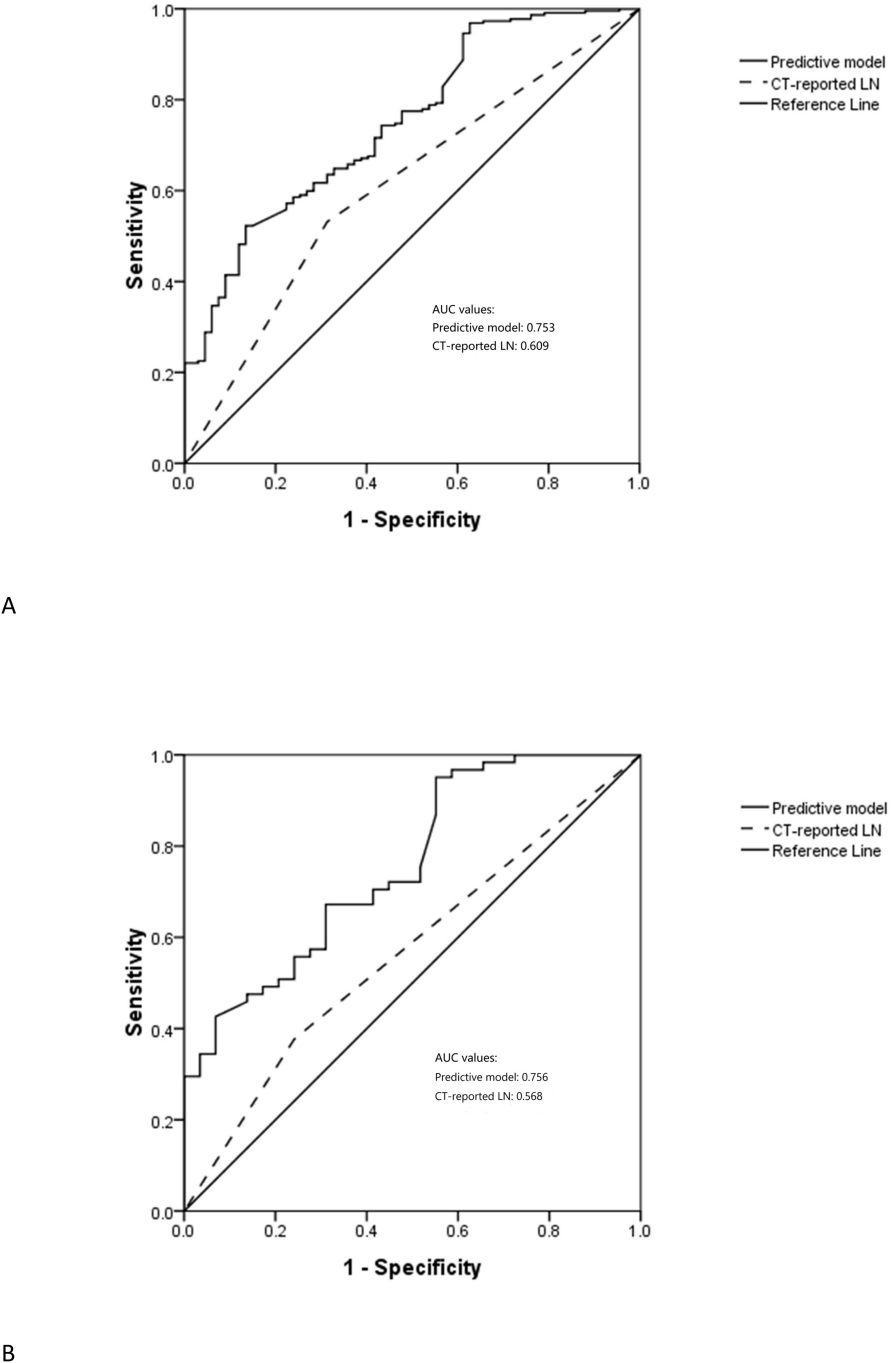
The ROC of predictive model in the **(A)** training and **(B)** test sets.

The respective AUC values for the PPM and CT-reported LN status of 0.753 and 0.609 differed significantly (P < 0.001) (**Figure 2A**).

### PPM validation

In the testing cohort, 29 (32.2%) and 61 (67.8%) patients were respectively diagnosed as negative and positive for LNM (**Table 1**). The AUC values for the PPM and CT-reported LN status differed significantly when clinical data from the testing cohort were included in the PPM (0.756 vs. 0.568, P < 0.001) (**Figure 2B**).

### Evaluation of model clinical utility

**Figure 3:**
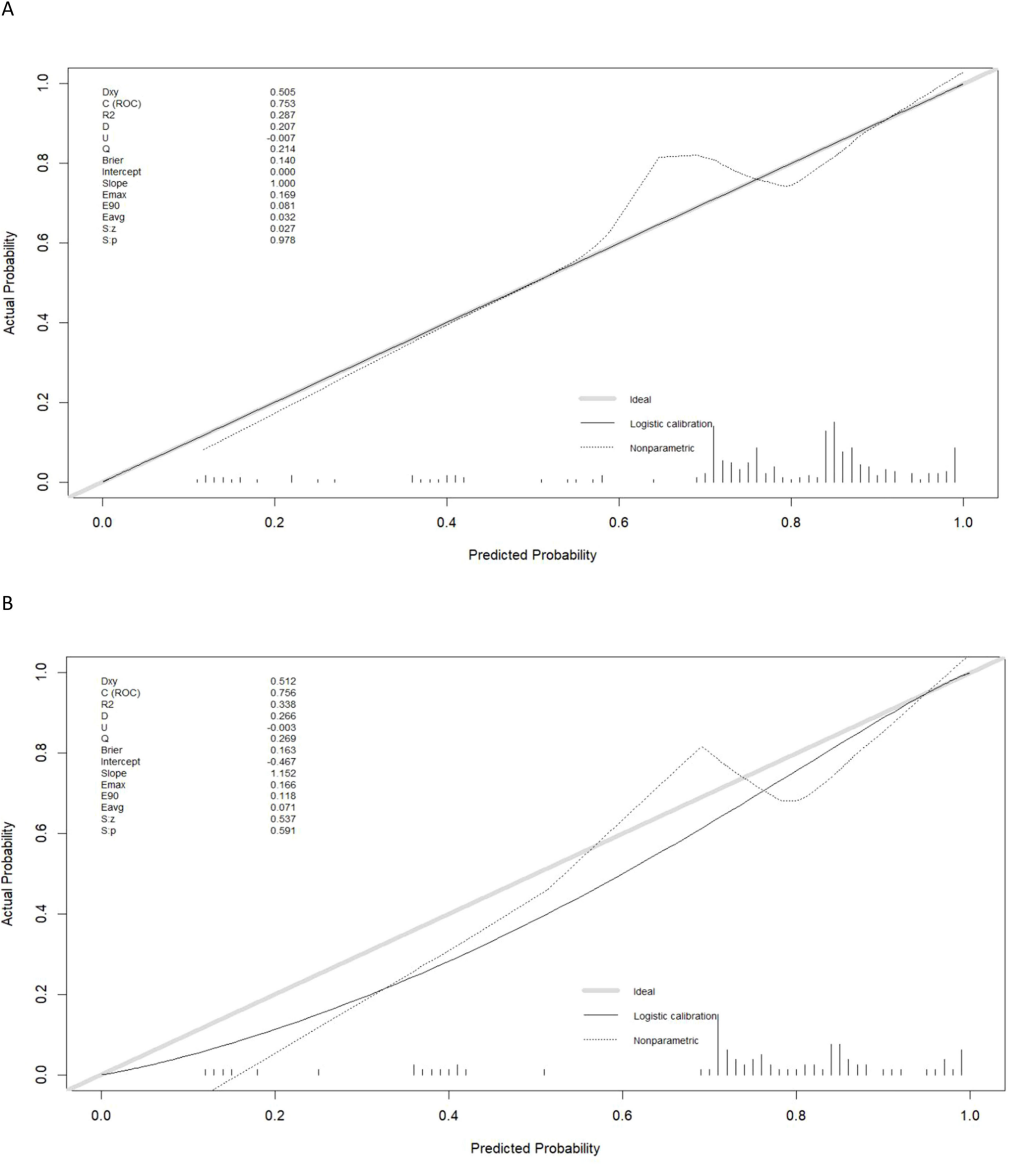
The calibration curve of predictive model in the **(A)** training and **(B)** test sets.

**Figure 4:**
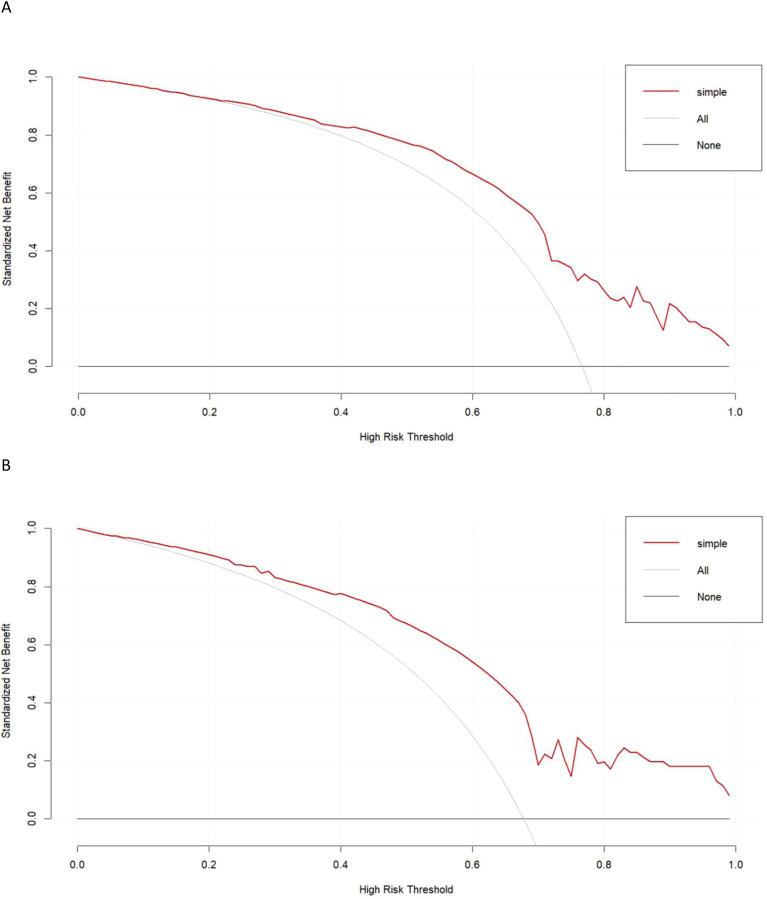
The decision curve of predictive model in the **(A)** training and **(B)** test sets.

Calibration curves generated for the training and testing cohorts revealed good consistency between actual and expected LNM status for these GC patients (**Figures 3A, B**). In all the training and testing cohorts, decision curve analyses showed a significant net clinical benefit associated with the PPM. The risk criteria for each of these two groups were 0.2-1.0 and 0.07-1.0, respectively (**Figures 4A, B**).

## Discussion

In this study, a PPM was developed and validated for the preoperative evaluation of the LNM status of patients with GCC. This model incorporated CT imaging, gastroscopy, and tumor marker-related findings, and yielded high AUC values in both the training (0.753) and testing (0.756) cohorts, with these AUC values being in line with those reported previously (0.716-0.794) (14, 15). In both investigated patient groups, the PPM showed considerably higher AUC values than those associated with CT-reported LN status. These findings suggested that PPM can accurately predict GC patients’ LNM status while also reliably guiding healthcare decision-making for these patients. Furthermore, the diagnostic usefulness of this model was greater than that of CT-reported LN status alone.

LN status as determined by CT scanning is regarded as a direct indicator of LNM status (4). As expected, CT-reported LN status was verified as an LNM-related risk factor in this investigation (P = 0.044). However, LN size cannot fully explain a given patient’s LNM status (16–18). In early GC patients, for instance, the sensitivity levels for CT-reported LN status were only between 4% and 34% in past studies (19, 20). The primary rationale behind this conclusion is that LNs may also appear large due to inflammation or reactivity, while in some instances, typically sized LN can be metastatic (1).

In cases of both LNM and distant metastasis, the biological features of the underlying primary tumor are linked to these forms of disease progression. Indeed, tumor invasion depth was found to be associated with LNM risk in this study, as was cT3 status (P = 0.001). Although the relationship between cT2 status and LNM was not statistically significant, there was a notable trend (P = 0.067) that supported the connection between deeper invasion and LNM. Previous research has also indicated that characteristics such as the existence of ulcers, lower differentiation of cells, and longer tumor length have been associated with LNM (15, 18). Although they may suggest a high degree of GC malignancy, they were not directly linked to the likelihood of LNMcurrent investigation. Based on these findings, it appears that local invasion is the primary component associated with GC migration.

LNM may contribute to tumor marker level abnormalities. In GC, preoperatively elevated serum levels of CA199 and CEA were significantly related to LNM and may offer utility as predictors of such metastasis (21). With respect to clinical features, higher CA199 and CEA levels tend to be associated with greater tumor invasiveness and metastasis (22). When diagnosing LNM in GC, Ding et al. (15) found that CA199 and CEA levels were significantly effective. In the current investigation, CA199 level was consistently linked to the incident of LNM in GC patients.

Furthermore, a PPM and nomogram were developed using the risk variables. The nomogram has several significant benefits. It enables the establishment of the risk levels that are ranked and the weights assigned to each element. Furthermore, it permits the prediction of the likelihood of LNM by comparing patient risk scores to a predetermined cut-off. Finally, the nomogram can make it possible to compute risk ratings and the corresponding predictive probabilities quickly.

This study is subject to some limitations. For one, these analyses were retrospective in nature such that they face a high potential for bias. Additionally, reviewers’ experience may influence the CT and gastroscopy assessments, contributing to a further risk of bias. Moreover, the study sample size was not large, precluding the identification of significance for factors previously reported to be predictive such as ulcer presence, poorer differentiation, or longer tumor length. Additional large-scale follow-up will thus be essential. Last, the “CT-reported LN” section indicated that the testing cohort had a lower positive predictive value compared to the training cohort. This discrepancy may be caused by selection bias, different CT parameters in different centers, and unbalanced sample size in training and testing cohorts.

## Conclusion

In conclusion, these analyses provide clear evidence in support of the diagnostic value of the PPM for the prediction of preoperative LNM status in patients with GC since this model was able to overcome analyses of CT-reported LN status alone in terms of diagnostic utility.

## Data Availability

The original contributions presented in the study are included in the article/**Supplementary Material**. Further inquiries can be directed to the corresponding author/s.
