# Supplementary material for: Preoperative predictive model for the probability of lymph node metastasis in gastric cancer: a retrospective study
Source: Front Oncol. 2024 Sep 27;14:1473423. doi: 10.3389/fonc.2024.1473423 (PMC11466724; doi:10.3389/fonc.2024.1473423)
Supplement: Supplementary file 1 [file DataSheet1.doc]

After the non-enhanced abdominal CT scan, the patients were intravenously injected with 1.5 mL/kg of iodinated contrast medium (Jiangsu Hengrui Pharmaceuticals Co.,Ltd, Jiangsu, China) at a flow rate of 3.0 mL/s by an automatic pump syringe. After the contrast agent injection starts, when the contrast agent concentration reached 100 Hu, the imaging after 8 seconds is the arterial phase, the imaging at 21 seconds after the arterial phase imaging is the venous phase, and the imaging at 90 seconds after the venous phase imaging is the delayed phase. The parameters of the CT scan were as follows: tube voltage 120 kV, tube current 150 - 300 mA, matrix 512 × 512, rotation time 0.5 seconds, pitch 1.0, and images were reconstructed with section thicknesses of 2 mm.

Table supplemental 1 The inter-observer agreement.

|  | ICC/Kappa value |
| --- | --- |
| Maximum tumor diameter |  |
| Training cohort | 0.997 |
| Test cohort | 0.995 |
| CT-reported LN status |  |
| Training cohort | 0.965 |
| Test cohort | 0.902 |

ICC: Intraclass Correlation Coefficient.
